# Substantial improvement of perovskite solar cells stability by pinhole-free hole transport layer with doping engineering

**DOI:** 10.1038/srep09863

**Published:** 2015-05-18

**Authors:** Min-Cherl Jung, Sonia R. Raga, Luis K. Ono, Yabing Qi

**Affiliations:** 1 Energy Materials and Surface Sciences Unit (EMSS), Okinawa Institute of Science and Technology Graduate University (OIST), 1919-1 Tancha, Onna-son, Okinawa, 904-0495, Japan

## Abstract

We fabricated perovskite solar cells using a triple-layer of *n-*type doped, intrinsic, and *p*-type doped 2,2′,7,7′-tetrakis(N,N′-di-p-methoxyphenylamine)-9,9′-spirobifluorene (spiro-OMeTAD) (*n*-*i*-*p*) as hole transport layer (HTL) by vacuum evaporation. The doping concentration for *n*-type doped spiro-OMeTAD was optimized to adjust the highest occupied molecular orbital of spiro-OMeTAD to match the valence band maximum of perovskite for efficient hole extraction while maintaining a high open circuit voltage. Time-dependent solar cell performance measurements revealed significantly improved air stability for perovskite solar cells with the *n*-*i*-*p* structured spiro-OMeTAD HTL showing sustained efficiencies even after 840 h of air exposure.

Recently, perovskite solar-cells have shown a rapid rising trajectory of efficiencies exceeding 19%.^1^ Such cells are advantageous because of easy fabrication and inexpensive raw materials.^2^,^3^,^4^,^5^,^6^ Perovskite thin films such as CH_3_NH_3_PbX_3_ (X = Cl, Br, I) can be formed as a result of reaction between CH_3_NH_3_X (X = I or Br) and PbX_2_ (X = I, Br, or Cl) using solution-based or vapor-based methods.^4^,^5^,^7^,^8^,^9^ A wide variety of strategies have been developed to achieve high efficiencies.^10^,^11^,^12^,^13^,^14^,^15^,^16^ It has been reported that perovskites show charge carrier mobility of ~10 cm^2^/V·s and long lifetime.^17^ The widely used hole transport layer (HTL) for these perovskite solar cells is spin coated 2,2′,7,7′-tetrakis(N,N′-di-p-methoxyphenylamine)-9,9′-spirobifluorene (spiro-OMeTAD) with 4-*tert*-butylpiridine (t-BP) and lithium *bis*-(trifluoromethylsulfonyl)imide salt (LiTFSI).^4^,^5^ A recent study from our group reveals that these as-prepared spin coated films of spiro-OMeTAD show a high density of pin-holes (Figure S1).^18^ The formation of pinholes is characteristic from the spin-coating procedure of the spiro-OMeTAD compound dissolved in chlorobenzene based on atomic force microscopy (AFM) measurements. Cross-sectional scanning electron microscopy (SEM) measurements reveal that these pinholes are observed to form channels across the film thickness (~240 nm).^18^ These pin-holes can cause the instability problem of perovksite solar cells, which is likely the reason why many perovskite solar-cell using spin coated spiro-OMeTAD films as HTL showed rapidly reduced efficiencies when exposed to air. On the other hand, spiro-OMeTAD films prepared by vacuum evaporation do not show pinholes.^19^ Therefore, vacuum evaporation is suitable to prepare pinhole-free spiro-OMeTAD films, which may solve this problem. However, when vacuum evaporated undoped (i.e. intrinsic) spiro-OMeTAD was used as HTL, the perovskite solar cell showed an extremely low efficiency of 0.7% (Figure S2 and Table S1) possibly due to the poor conductivity of undoped spiro-OMeTAD.^19^ Therefore, doping is needed to ensure efficient hole transport in vacuum evaporated HTL.^20^,^21^,^22^,^23^ On the other hand, a simple bi-layer of intrinsic and *p*-type doped HTL is not sufficient, as evidenced by the still relatively low efficiency (5.9%) of a perovskite solar cell with 30-nm thick undoped spiro-OMeTAD / 20-nm thick *p*-type doped spiro-OMeTAD by tetrafluorotetracyanoquinodimethane (F4-TCNQ) (Figure S2 and Table S1).

In this manuscript, we fabricated perovskite solar cells using a triple-layer of *n*-type doped, intrinsic and *p*-type doped 2,2′,7,7′-tetrakis(N,N′-di-p-methoxyphenylamine)-9,9′-spirobifluorene (spiro-OMeTAD) (*n*-*i*-*p*) as hole transport layer (HTL). Dopants used for this work are *n*–type dopant decamethylcobaltocene (DMC) and *p*–type dopant F4-TCNQ. We measured the energy level of highest occupied molecular orbital (HOMO) for each doping layer using *in-situ* ultraviolet photoemission spectroscopy (UPS). On the basis of the energetics studies, we identified the optimal *n*-type doping concentration that can match the HOMO level with the valence band maximum (VBM) of perovskite. In general, the *n-i-p* structure is used in organic based device application to improve its carrier performance.^24^ The *n-i-p* structure makes a smoother transition in the HOMO energy steps conducting efficiently the hole carriers and minimizes electron-hole recombination processes.^24^ In addition, the intrinsic layer acts as a barrier layer between the *n*- and *p*-type materials avoiding dopants inter-diffusion. We measured the solar cell performance as a function of storage time in air, and found that the efficiencies for perovskite solar cells with vacuum evaporated *n*-*i*-*p* structured spiro-OMeTAD HTL was 3.6% when freshly fabricated, increased to 9.1% after storage in air for 310 h, and retained a similar level of efficiency even after storage in air for 840 h. This stable behavior is in sharp contrast to the reference cells with spin coated doped spiro-OMeTAD according to the widely used recipe, which showed high efficiencies of 12.5% in average (or 13.5% for the best performing device), but rapidly decreased to 4.8% after storage in air for 360 h. We propose that our method of the vacuum evaporated *n*-*i*-*p* structured spiro-OMeTAD HTL can be a good solution to the fabrication of perovskite solar cells with enhanced air stability.

## Results

**Figure 1:**
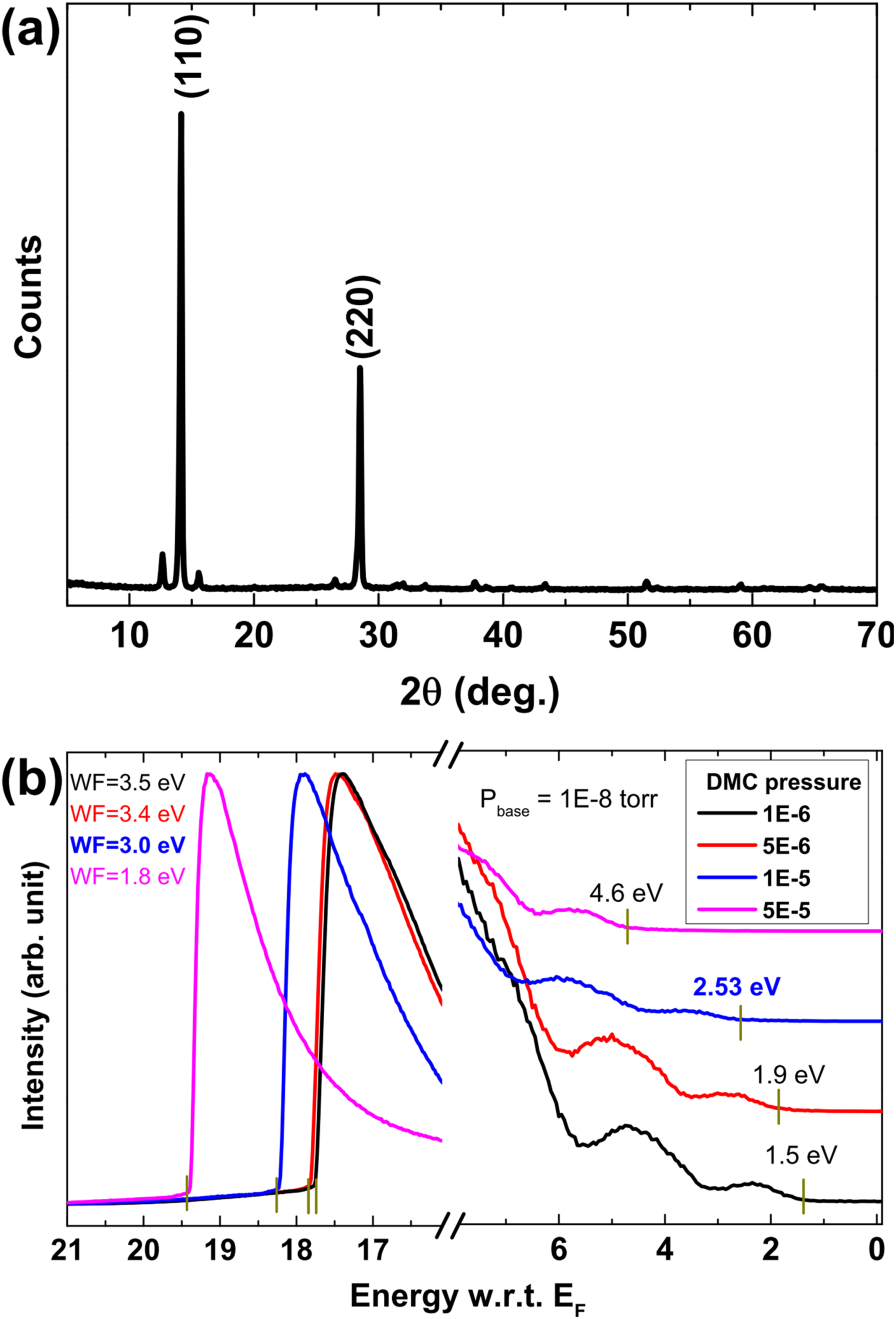
(a) X-ray diffraction of the spin coated perovskite film showing (110) and (220) peaks. (b) UPS spectra on *n*-type doped spiro-OMeTAD with different DMC vapor pressures.

XRD measurements on our perovskite thin films showed typical perovskite (110) and (220) peaks at 14.2 ° and 28.5 °, respectively (Fig. 1a).^4^,^5^ To find the optimal condition of DMC vapor pressure, we performed the spiro-OMeTAD deposition with different DMC vapor pressure from 1.0 × 10^–6^ to 5.0 × 10^–5^ *Torr*. Based on UPS measurements, work functions were 3.5, 3.4, 3.0, and 1.8 eV under the working pressures of 1.0 × 10^–6^, 5.0 × 10^–6^, 1.0 × 10^–5^, and 5.0 × 10^–5^ *Torr*, respectively (Fig. 1b). Also, the HOMO leading edge for each pressure was measured to be 1.5, 1.9, 2.5, and 4.6 eV below the Fermi level (*E_F_*). These measurements confirm the *n*-type doping behavior using DMC as the dopant and the possibility to tune the HOMO level of spiro-OMeTAD with respect to the Fermi level by varying the DMC vapor pressure.^20^,^24^

**Figure 2:**
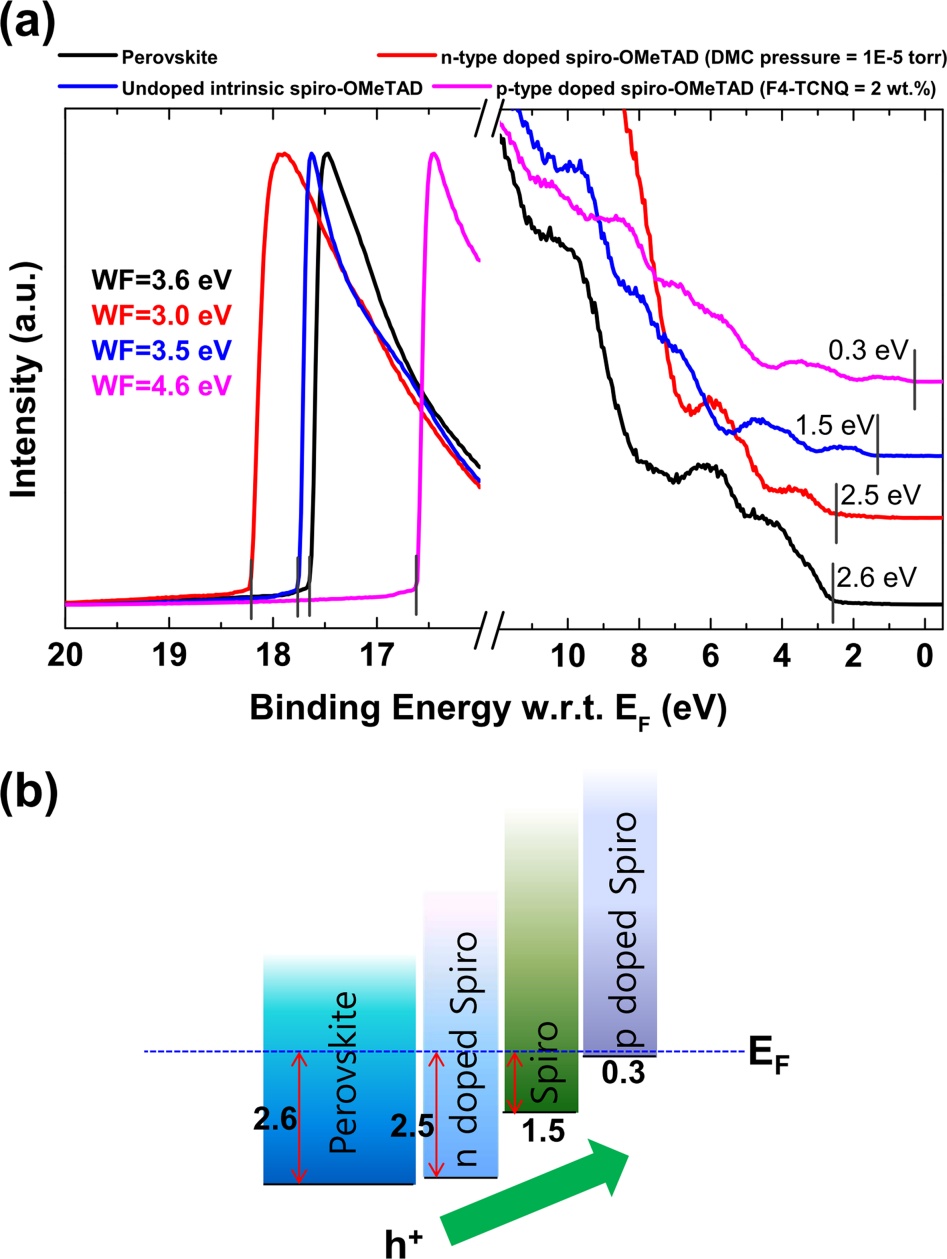
(a) UPS spectra of perovskite, *n*-type doped spiro-OMETAD (DMC vapor pressure ~10^–5^ *Torr*), undoped intrinsic spiro-OMeTAD, and *p*-type doped spiro-OMeTAD (F4-TCNQ ~2 wt.%). (b) Energy level alignment diagram extracted from the UPS results.

Furthermore, we performed *in situ* UPS measurements on *n*-type doped, intrinsic, and *p*-type doped spiro-OMeTAD by depositing these layers sequentially on a solution prepared perovskite film on a FTO substrate pre-coated with a TiO_2_ compact layer. Figure 2(a) shows UPS spectra of perovskite, *n*-type doped, intrinsic, and *p*-type doped spiro-OMeTAD. The valence band maximum of perovskite was measured to be 2.6 eV below E*_F_*. An optimal DMC vapor pressure of 1.0 × 10^–5^ *Torr* was identified, which enabled the matching between the valence band maximum of perovksite and the HOMO level of DMC doped spiro-OMeTAD (Fig. 2b).^20^,^23^ In the case of intrinsic spiro-OMeTAD, this layer is used to minimize the inter-diffusion of *n-* and *p*-type dopants. For this layer, the HOMO level was found to be at 1.5 eV below *E_F_*, which is consistent with a previous report.^19^ The HOMO level of F4-TCNQ (2 wt.%) doped spiro-OMeTAD was determined to be 0.3 eV below *E_F_*, which agrees well with the *p*-type doping effect.^21^,^22^ Based on these UPS results, we can precisely determine the energy diagram for this solar cell, which is expected to have a strong correlation with solar cell performance (Fig. 2b). For instance, the built-in potential represented by the staircase change of the HOMO levels across the *n*-type, *i*-type, and *p*-type layers in Fig. 2b is expected to enhance the hole carrier extraction efficiency.^24^ Small amounts of F4-TCNQ dopant induce a high doping efficiency in the host spiro-OMeTAD molecules pinning the Fermi level to its HOMO level. Thus, devices with only *p*-type layers will generate a large gap (~2.2 eV) between *p*-doped spiro-OMeTAD HOMO and perovskite VBM is not optimum for efficient hole extraction. Minimizing charge carrier injection barriers and extraction losses at interfaces is critical for optimizing solar cell device performance.

## Discussion

**Figure 3:**
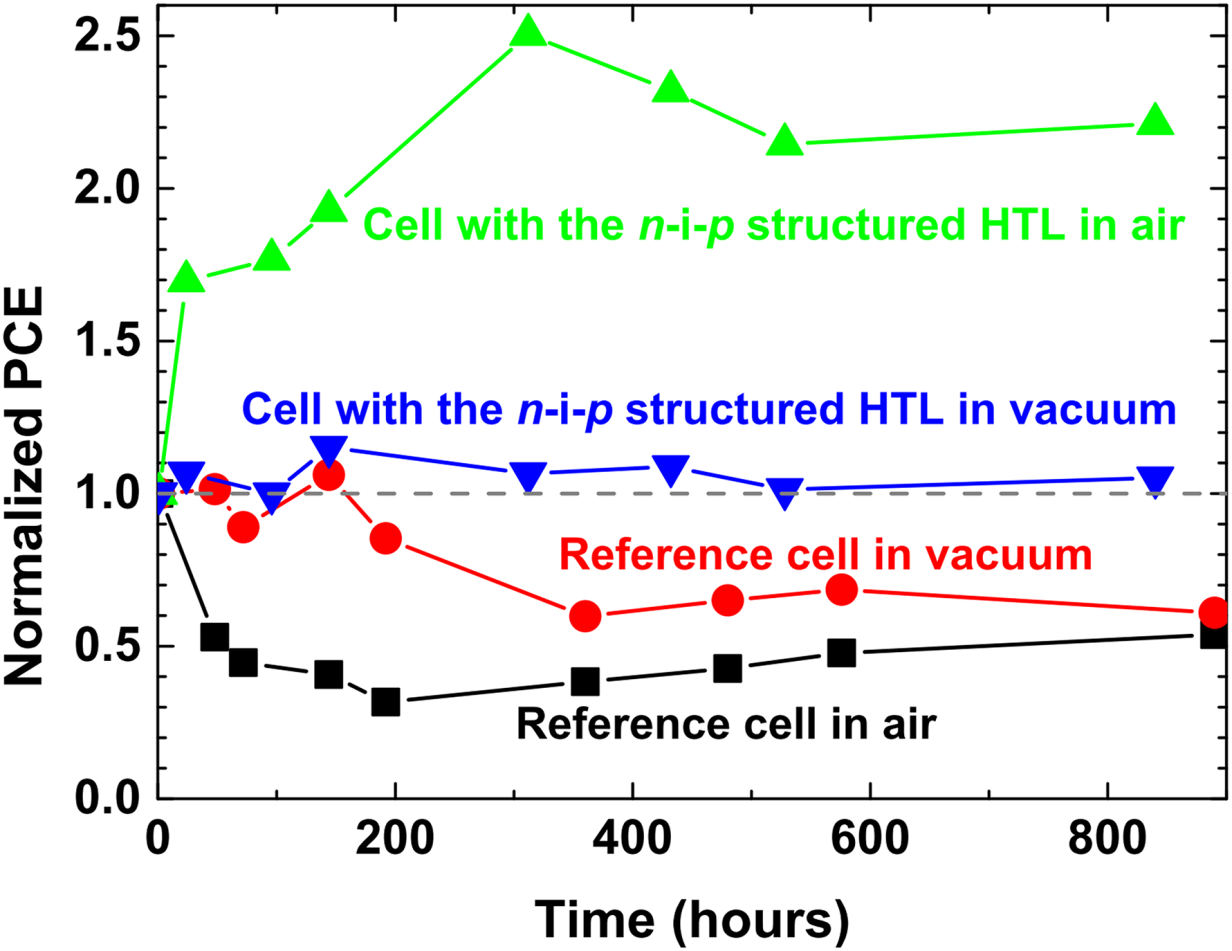
Plot of normalized PCE as a function of time. The average value of PCE for the freshly-prepared solar cells is used as the normalization reference value (i.e. for the four types of samples, their initial PCE values are all normalized to 1). All measurements have been done after pre-illuminating the solar cells for 40 s under the open circuit condition. The *j-V* scan was performed from 1.1 V to 0 V at a scan rate of 0.2 V·s^–1^. After 800 h, the reference samples stored in air and in vacuum showed 50~60% of their initial efficiencies. However, the two cells with the *n-i-p* structure HTL did not show any sign of degradation after 800 h under both storage conditions. Adapted with permission from Hawash, Z.; Ono, L. K.; Raga, S. R.; Lee, M. V.; Qi, Y. B. *Chem. Mater.* **2015**, *27*, 562. Copyright 2015 American Chemical Society.

To study the stability of solar cell devices, we measured the solar cell performance with the time evolution up to 890 h in air and in high vacuum with the pressure of 10^–6^ *Torr* (Fig. 3 and Table 1). We prepared two reference samples (total 12 solar cell devices) with standard spin coated doped spiro-OMeTAD as HTL and two other samples (total 12 devices) with vacuum evaporated *n*-*i*-*p* structured spiro-OMeTAD HTL. After approximately 300 h, all samples roughly stabilized to a constant PCE value. The PCEs of the two reference samples decreased and stabilized to a value close to 50% for the air-stored reference sample and 60% for the vacuum-stored reference sample. The main reason for the lower efficiency is a lower photocurrent and a large decrease in FF (Figure S3b and c). The decrease in *j_sc_* and *FF* is likely caused by perovskite layer degradation, which can be induced by (1) air molecules (O_2_, H_2_O, etc.) migration and interaction with perovskite via the pinholes in the spin coated spiro-OMeTAD HTL and/or (2) out-diffusion of mobile ions in the perovskite film via the pinholes in the spin coated spiro-OMeTAD HTL. For the samples with the vacuum evaporated *n*-*i*-*p* structured HTL, on the other hand, the efficiency obtained after 840 h (35 days) is even larger than that measured on the fresh devices, more pronounced for the air-stored sample. The PCE improvement is primarily due to the increase of *V_oc_* and *FF* from initial low values to values that are roughly the same as those for the freshly prepared reference devices. We attribute it to the doping effects of the intrinsic spiro-OMeTAD layer by either the diffused F4-TCNQ, DMC dopants or air molecules, leading to reduced series resistance.^25^ We observed a similar improvement for solar cells prepared with undoped intrinsic spiro-OMeTAD after 5 days storage in a N_2_ glove box with a few hours of exposure in ambient air with a relative humidity of ~50% (Figure S4). Finally, the photocurrent for the two samples with the vacuum evaporated *n*-*i*-*p* structured HTL remained almost constant over the 840 h of study (Table 1). A constant *j_sc_* indicates a good stability of the perovskite layer that is protected from air exposure by the vacuum evaporated pinhole-free HTL. This is further corroborated by AFM (Figure S1) and SEM,^18^,^19^ which the vacuum prepared spiro-OMeTAD films showed absence of pinholes. The amorphous nature of spiro-OMeTAD films and its high stability (glass-transition temperature T_g_ = 121 °C)^19^ are expected to serve as gas barrier layer for the underneath active perovskite material.

The efficiencies of the *n-i-p* HTL samples stored in air increased during the first ~300 h in our device life-time tests. The architecture of our cells comprising of FTO/c-TiO_2_/perovskite/*n*-*i*-*p* HTL/Au is complex and convoluted physico-chemical processes taking place within the layers are present to explain the non-monotonic behavior in PCE. The observed efficiency enhancement may be attributed due to dopants redistribution^18^ induced by the ambient air exposure. This can be correlated also by the observed improvement in Voc (Figure S3) meaning that gradual changes in energy levels take place within the *n*-*i*-*p* and/or with the underneath perovskite layers upon air exposure.^18^ Further investigation is needed to determine unambiguously the origin of such efficiency enhancement.

In summary, organometal halide perovskite based solar cells using a triple-layer of *n*-type doped, intrinsic and *p*-type doped (spiro-OMeTAD) as HTL show substantially improved air stability. The doping concentration for *n*-type doped spiro-OMeTAD was optimized to match the spiro-OMeTAD HOMO with the valence band maximum of perovskite for efficient hole extraction while maintaining a relatively high open circuit voltage. It is expected that vacuum evaporated spiro-OMeTAD with doping engineering can be a promising route for fabricating high-efficiency high-stability perovskite solar cells.

## Methods

### The sample preparation

The patterned fluorine-doped tin oxide glass (FTO Pilkington, 7 Ω/□) was prepared by HCl and Zn powder and cleaned. On this substrate, we deposited 100 nm thick compact layer of TiO_2_ by spray pyrolysis with a precursor solution of acetylacetone, Ti (IV) isopropoxyde and anhydrous ethanol (3:3:2) and then performed post-annealing at 480 °C on a hotplate. MAI and PbCl_2_ (Sigma-Aldrich) were dissolved in *N,N*-dimethylformamide at a 2.5:1 molar ratio with a concentration of 2.2 M MAI and 0.88 M PbCl_2_. Perovskite solution was spin coated on the compact TiO_2 _at 2000 rpm for 45 sec followed by a thermal annealing on the hotplate for 45 min in the glove box (< 0.1 ppm O_2_ and H_2_O). To make a reference sample, we used a typical spin-coating method (2000 rpm for 60 s) with a solution consisting of 59 mM of spiro-OMeTAD, 172 mM 4-*tert*-butylpiridine (tBP) and 32 mM lithium *bis*-(trifluoromethylsulfonyl)imide salt in chlorobenzene to prepare HTL. To fabricate *n*-type doped spiro-OMeTAD by vacuum evaporation, we placed 5 mg of DMC powder in a glass ampule that is connected with an all-metal leak valve. The filling of DMC to the ampule was performed in a N_2_ glovebox to avoid the air exposure induced oxidation of DMC. Then we installed the ampule with DMC powder to the vacuum chamber. To evaporate DMC powder, we used a heating tape and heated the ampule to ~100 °C. The base pressure of vacuum chamber was 1.0 × 10^–8^ *Torr*. We varied the DMC vapor pressure from 1.0 × 10^–6^ to 5.0 × 10^–5^ *Torr* to find an optimal DMC doping concentration that can match the HOMO level of the *n*-type doped spiro-OMeTAD layer to the valence band maximum of perovskite. Under the optimal DMC vapor pressure of 1.0 × 10^–5^ *Torr*, we performed *n*-type doped spiro-OMeTAD evaporation with the deposition rate of 0.5 Å/s measured by a quartz crystal microbalance (INFICON Co.). When the deposition of the *n*-type doped spiro-OMeTAD was completed, we deposited undoped spiro-OMeTAD (i.e., intrinsic) in the same chamber. To fabricate *p*-type doped spiro-OMeTAD, we moved the sample to a second chamber and performed co-evaporation with F4-TCNQ (deposition rate = 0.1 Å/s corresponding to 2 wt.%) and spiro-OMeTAD (deposition rate = 0.5 Å/s).

### Measurements of thickness, structural phase, energy levels, solar cell performance

On the basis of atomic force microscopy (MFP-3D Asylum Research) measurement results, the thicknesses of *n*-type doped spiro-OMeTAD, intrinsic spiro-OMeTAD, and *p*-type doped spiro-OMeTAD were 30, 20, and 30 nm, respectively. Lastly, 60 nm-thick gold contacts were deposited by thermal evaporation. To confirm the crystalline structure of perovskite, we used x-ray diffractometer (D8 Discover, Bruker Corporation). We also performed *in-situ* UPS measurements on each of the three layers of the *n*-*i*-*p* structured HTL using the He I (21.2 eV) discharging lamp and EA125 energy analyzer with single channeltron (Focus and Omicron Nanotechnology). The Fermi edge of a gold film deposited on a high *n*-doped Si substrate (0.011 ~0.015 Ω cm) was used to determine the *E_F_* position and the instrumental resolution. Current-voltage device characteristics were measured by applying an external potential bias under standard 1 sun AM1.5 simulated solar irradiation (100 mW/cm^2^, Newport Oriel Sol1A) and measuring the photocurrent generated (Keithley 2420 source meter). The final measured efficiencies were prone to variations from sample-to-sample preparation conditions due to the complexity (fabrication with multi step processes) of the solar cell architecture (FTO/c-TiO_2_/perovskite/*n*-*i*-*p* HTL/Au). The results reported in our work correspond to statistical analysis based on at least six devices. Thus, the reported trends are not influenced by the sample-to-sample variation due to a relatively large number of devices prepared.

## Author Contributions

Y.B.Q. conceived the idea, designed the experiments and supervised the project. M.-C.J., S.R.R., and L.K.O performed a major portion of sample preparation, XRD, AFM, UPS, and solar cell performance measurements. All authors discussed the results, performed data analysis and explanation, wrote the manuscript and revised it.

## Additional Information

**How to cite this article**: Jung,M.-C., Raga, S.R., Ono, L.K.& Qi,Y. Substantial improvement of perovskite solar cells stability by pinhole-free hole transport layer with doping engineering. *Sci. Rep.* **5**, 9863; doi: 10.1038/srep09863 (2015).

## Supplementary Material

Supplementary Information Supplementary Information

## Figures and Tables

**Table 1:** Photovoltaic parameters extracted from the *j-V* curves of the solar cells, measured at 1 sun illumination conditions (100 mW/cm^2^) for the fresh cells, and after storage in air or in vacuum for 430–480 h and 840–890 h. The values correspond to the best efficiency device of the batch at the time when it was measured. The values in brackets correspond to the average values from all the devices in a batch and the corresponding standard deviation.

Sample	Time (hours)	*V_oc_* (V)	*j_sc_* (mA cm^–2^)	FF (%)	PCE (%) (Avg.)
	fresh	0.967	23.1	60.3	13.5
		(0.973 ± 0.014)	(22.0 ± 2.0)	(58.0 ± 4.4)	(12.5 ± 1.9)
**Reference cell in air**	+480 h	0.908	17.6	45.8	7.3
		(0.897 ± 0.047)	(13.9 ± 3.3)	(42.2 ± 3.5)	(5.3 ± 1.5)
	+890 h	0.925	19.4	42.1	7.5
		(0.903 ± 0.045)	(19.2 ± 2.5)	(41.8 ± 2.6)	(6.7 ± 1.0)
	fresh	0.914	19.8	61.4	11.1
**Reference cell in vacuum**		(0.894 ± 0.018)	(19.8 ± 0.6)	(54.8 ± 3.2)	(9.3 ± 0.8)
	+480 h	0.885	14.0	52.6	6.5
		(0.878 ± 0.012)	(15.9 ± 2.7)	(44.2 ± 6.0)	(6.1 ± 0.6)
	+890 h	0.896	18.3	35.1	5.7
		(0.904 ± 0.010)	(17.7 ± 2.0)	(35.5 ± 3.6)	(5.7 ± 0.6)
	fresh	0.706	17.8	37.9	4.8
		(0.711 ± 0.170)	(15.9 ± 1.4)	(34.4 ± 2.7)	(3.6 ± 1.1)
	+430 h	0.980	17.8	55.7	9.7
**Cell with *n*-*i*-*p* HTL in air**		(0.904 ± 0.045)	(17.2 ± 1.0)	(54.7 ± 2.5)	(8.4 ± 0.9)
	+840 h	0.887	17.6	53.2	8.3
		(0.885 ± 0.021)	(16.4 ± 1.0)	(53.9 ± 1.4)	(8.0 ± 0.5)
	fresh	0.819	19.4	55.7	8.9
		(0.778 ± 0.037)	(19.3 ± 0.3)	(48.9 ± 3.8)	(7.4 ± 0.9)
**Cell with *n*-*i*-*p* HTL in vacuum**	+430 h	0.892	18.1	55.3	8.9
		(0.834 ± 0.072)	(18.4 ± 0.5)	(51.8 ± 1.9)	(8.1 ± 0.9)
	+840 h	0.841	18.6	57.5	9.0
		(0.817 ± 0.021)	(18.7 ± 0.3)	(51.5 ± 3.0)	(7.8 ± 0.6)
