# Supplementary material for: Substantial improvement of perovskite solar cells stability by pinhole-free hole transport layer with doping engineering
Source: Sci Rep. 2015 May 18;5:9863. doi: 10.1038/srep09863 (PMC4434888; doi:10.1038/srep09863)
Supplement: Supplementary Information [file srep09863-s1.docx]

Supplementary information

**Substantial improvement of perovskite solar cells stability by pinhole-free hole transport layer with doping engineering**

Min-Cherl Jung, Sonia R. Raga, Luis K. Ono, and Yabing Qi^*^

*Energy Materials and Surface Sciences Unit (EMSS), Okinawa Institute of Science and Technology Graduate University (OIST), 1919-1 Tancha, Onna-son, Okinawa, 904-0495, Japan*

*Correspondence: Professor Yabing Qi, Energy Materials and Surface Sciences Unit (EMSS), Okinawa Institute of Science and Technology Graduate University (OIST), 1919-1 Tancha, Onna-son, Okinawa, 904-0495, Japan, Tel: +81-998-966-8435, Email: [Yabing.Qi@OIST.jp](mailto:Yabing.Qi@OIST.jp)

**Surface morphology of spin coated and vacuum evaporated spiro-OMeTAD films**


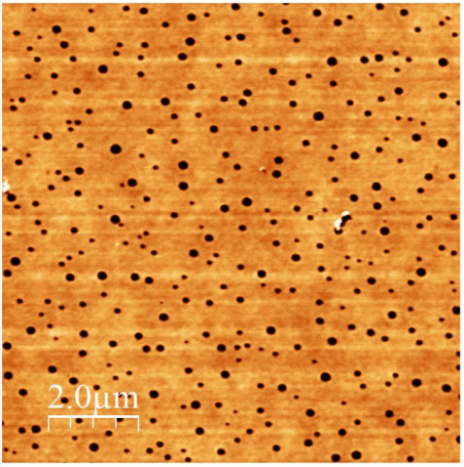

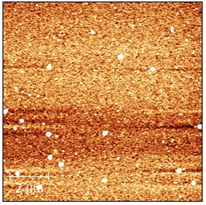


**(b)**

**(a)**

Figure S1. Tapping mode atomic force microscopy topography images of (a) the spin coated spiro-OMeTAD with t-BP and LiTFSI, (b) the vacuum evaporated F4-TCNQ (2 wt.%) doped spiro-OMeTAD film. The scan range of both images is 10 × 10 µm^2^. The diameter of pinholes is approximately 135 nm on average.

**The solar cell performance measurements**

Figure S2. Voltage versus current density plots for the perovskite reference cell using standard spin coated HTL (240-nm thick spin coated spiro-OMeTAD with t-BP and LiTFSI), the cell with vacuum evaporated *n-i-p* structured HTL (20-nm thick DMC doped spiro-OMeTAD \ 30-nm thick undoped spiro-OMeTAD \ 20-nm thick F4-TCNQ doped spiro-OMeTAD), the cell with undoped (i.e. instrinsic) spiro-OMeTAD (30-nm thick), and the cell with the *i-p* structured HTL (30-nm thick undoped spiro-OMeTAD / 20-nm thick F4-TCNQ doped spiro-OMeTAD). In the case of the cell with the *i-p* structured HTL, *V_oc_* is substantially lower than the cell with *n*-i-*p* structured HTL (See Table S1).

Table S1. Summary of photovoltaic parameters extracted from the j-V curves shown in Figure S2. The solar cell devices were measured at 1 sun illumination conditions (100 mW/cm^2^).

|  | ***V_oc_* (V)** | ***j_sc_* (mA/cm^2^)** | **FF (%)** | **PCE (%)** |
| --- | --- | --- | --- | --- |
| **Reference cell with spin coated HTL** | 0.967 | 23.1 | 60.3 | 13.5 |
| **Cell with *n*-*i*-*p* structured HTL** | 0.819 | 19.4 | 55.7 | 8.9 |
| **Cell with *i-p* structured HTL** | 0.651 | 16.5 | 54.7 | 5.9 |
| **Cell with undoped HTL** | 0.662 | 5.2 | 19.9 | 0.7 |

**Time evolution of the photovoltaic parameters extracted from *j-V* curves**


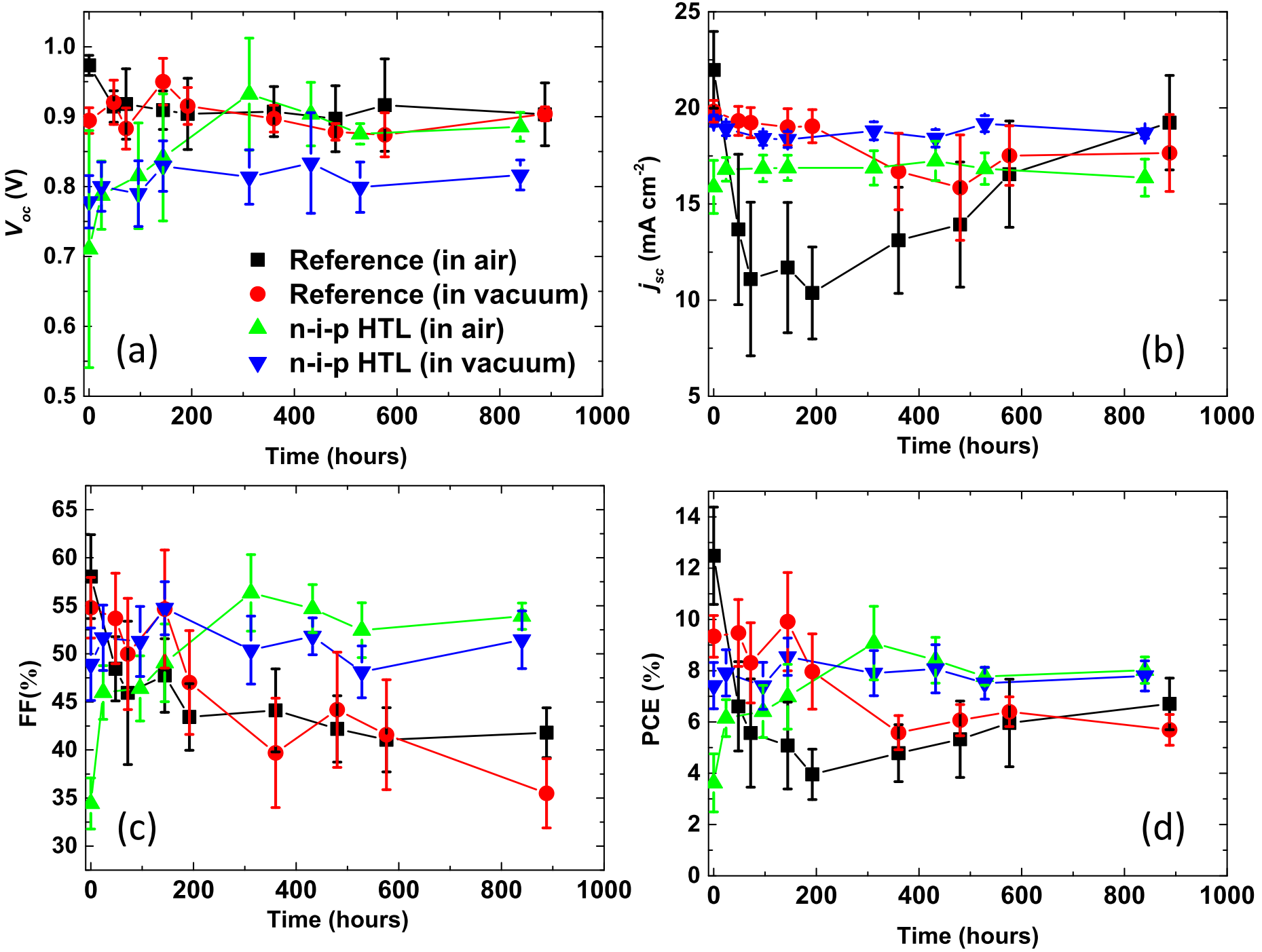


Figure S3. (a) *V_oc_*, (b) *j_sc_*, (c) *FF* and (d) PCE as a function of time. After 600 h, the reference cell stored in vacuum and the cell with the *n-i-p* structured HTL stored in vacuum were transferred from vacuum chamber to a N_2_ glove box for storage. *V_oc_* retained the original values roughly for all the cells except the cell with the *n*-*i*-*p* structured HTL in vacuum, which had the substantial increase in *V_oc_*. The photocurrent of the two cells with the *n*-*i*-*p* structured HTL showed high stability after 800 h storage under both conditions. In the case of fill factor (*FF*), the two reference cells degraded after 200 h and continued decreasing over time, compared to the stable FF of the two cells with the *n*-*i*-*p* structured HTL. Adapted with permission from Hawash, Z.; Ono, L. K.; Raga, S. R.; Lee, M. V.; Qi, Y. B. *Chem. Mater.* **2015**, *27*, 562. Copyright 2015 American Chemical Society.

**Solar cells with undoped spiro-OMeTAD**





Figure S4. *j-V* curves of a solar cell with vacuum evaporated undoped (i.e. intrinsic) spiro-OMeTAD. Black squares represent the *j-V* curve for the fresh device, and red triangles represent the *j-V* curve for the same device after 5-days storage in the N_2_ glove box (with a few hours of exposure in ambient air; relative humidity ~ 50 %). It was observed that the decrease in series resistances of the device caused fill factor (FF) to increase from 19 % to 34 %. Increased photocurrent is a result of enhanced conductivity of the spiro-OMeTAD HTL caused by the doping effect from ambient exposure.
